# Heterogeneous myocardial contraction detected by speckle tracking echocardiography in systemic lupus erythematosus is associated with complement protein C4: a cross-sectional study from a Swedish tertiary referral centre

**DOI:** 10.1007/s00296-025-05939-8

**Published:** 2025-08-06

**Authors:** Faria Shahab, Helene Zachrisson, Christina Svensson, Meriam Åström Aneq, Christopher Sjöwall, David Kylhammar

**Affiliations:** 1 Department of Rheumatology and Department of Biomedical and Clinical Sciences, Linköping University, Linköping, Sweden; 2 Department of Clinical Physiology and Department of Health, Medicine and Caring Sciences, Linköping University, Linköping, Sweden; 3 Wallenberg Centre for Molecular Medicine, Linköping University, Linköping, Sweden

**Keywords:** Systemic lupus erythematosus, Echocardiography, Complement system proteins, Cardiovascular diseases, Myocardium, Heart Failure

## Abstract

Although systemic lupus erythematosus (SLE) is associated with an increased risk of cardiovascular disease, conventional echocardiographic measures often fail to detect early myocardial dysfunction. This study assesses left ventricular mechanical dispersion (LVMD) and its associations with vascular pathology in patients with SLE, with the focus on complement proteins. A cross-sectional study was conducted on 55 patients with SLE, categorised into sub-groups with skin and joint involvement, lupus nephritis (LN), and antiphospholipid syndrome (APS), along with 31 age- and sex-matched healthy controls. Speckle tracking echocardiography was used to assess myocardial function, including global longitudinal strain (GLS) and LVMD. The vascular pathology of each subject was evaluated using carotid and central arteries ultrasound to assess the intima-media thickness (IMT) and atherosclerotic plaque burden. LVMD was significantly increased in patients with SLE compared with the controls (p < 0.001), with APS patients displaying the highest values (median, 51 ms vs. 36 ms in controls). GLS did not differ between the groups. APS and LN patients exhibited mild right ventricular impairment. Higher LVMD values correlated with increased IMT in the internal carotid artery (p = 0.04) and aortic arch (p < 0.001), as well as with increased atherosclerotic plaque burden (p = 0.04). In multivariable regression analysis, complement protein C4 was an independent predictor of LVMD, suggesting a link between overall systemic inflammation and myocardial dysfunction in SLE. Our findings highlight the importance of speckle tracking echocardiography in uncovering sub-clinical cardiac dysfunction in SLE and call for additional research on complement-directed therapies to prevent atherosclerotic inflammation.

**Supplementary Information:**

The online version contains supplementary material available at 10.1007/s00296-025-05939-8.

## Introduction

Systemic lupus erythematosus (SLE) is a systemic inflammatory disease that often involves several organ systems, including the cardiovascular system [1, 2]. In sharp contrast to several other rheumatic diseases, patients who are suffering from SLE have higher rates of morbidity and mortality related to cerebrovascular and cardiovascular diseases (CVD), and they frequently report poor quality of life with considerable pain, fatigue and debilitated well-being [3, 4]. The relative risk of myocardial infarction or stroke has been estimated as approximately 2–3-times higher than in the general population [5–7]. The highest relative risks have been reported for pre-menopausal women (8–50-fold higher risk), early during the course of SLE (< 1 year after diagnosis, 4–tenfold increased risk), and for patients with lupus nephritis (LN; 4–18-fold higher risk) [8–10].

Left ventricular (LV) dysfunction is related to an increased risk of cardiovascular disease [11]. Traditionally, LV systolic function has been evaluated through measurement of the left ventricular ejection fraction (LVEF) from conventional trans-thoracic echocardiographic images. A reduction in LVEF is, however, seen only in the more-advanced stages of cardiac disease, making it unsuitable for the detection of subtle contractile abnormalities at earlier stages. During the past few years, speckle tracking echocardiography (STE) has emerged as a method to detect more-subtle impairment of ventricular systolic function. The STE methodology enables a detailed analysis of ventricular deformation by tracking small pixels, i.e. speckles, in the echocardiographic image during the cardiac cycle [12]. The well-established STE measure of LV systolic function is global longitudinal strain (GLS), which quantifies the degree of LV longitudinal shortening during systole [13]. Measures of longitudinal strain also allow the determination of contraction heterogeneity through analysis of left ventricular mechanical dispersion (LVMD), which may be an even more-sensitive marker of LV dysfunction and increased CVD risk [14].

Recent studies have indicated that GLS is reduced in patients with SLE [15–18], and impaired GLS has been found to be associated with an increased frequency of adverse cardiovascular events in patients with SLE [19, 20]. There are also some reports of increased LVMD in patients with SLE [21, 22]. Nevertheless, SLE remains a heterogeneous disease, and previous studies have generally not distinguished between the three SLE sub-sets, i.e. SLE with skin and joint involvement only, LN, and antiphospholipid syndrome (APS). In addition, there is a lack of studies investigating the associations between sub-clinical cardiac dysfunction and vascular disease in patients with SLE.

Thus, the aims of the current study were to: (i) characterise, using STE, the left and right ventricular functions in a well-characterised cohort of patients with skin and joint involvement only, LN or APS; and (ii) investigate the associations between sub-clinical cardiac dysfunction and clinical characteristics, as well as concomitant vascular involvement, including large vessel atherosclerosis, and circulating inflammatory biomarkers, including complement proteins.

## Patients & methods

### Study population

The 60 subjects recruited to this cross-sectional study were already enrolled in the observational program KLURING (Swedish acronym for the *Clinical Lupus Register in North-Eastern Gothia* cohort), which continuously includes and follows patients diagnosed with SLE at the Department of Rheumatology, Linköping University Hospital since 2008 [23]. Fulfilment of either the 1982 American College of Rheumatology (ACR-82) or the 2012 Systemic Lupus International Collaborating Clinics (SLICC-12) classification criteria was required to be eligible [24, 25]. Patients above > 65 years of age were excluded due to a higher background risk of manifest atherosclerosis dependent on age, whereas patients below the age of 25 years were excluded due to an overall short duration of SLE [26]. All 60 subjects were examined by echocardiography, five of whom were subsequently excluded from this study due to prevalent heart disease (two with atrial fibrillation, one with heart failure and atrial fibrillation, one with ischaemic heart disease, and one who had a mitral valve prosthesis). Of the remaining subjects, 18 (32.7%) met the renal disorder ACR-82 criterion, i.e., LN in the absence of APS, 18 (32.7%) met the Sydney classification criteria for APS [27] in the absence of LN, and 19 (34.5%) had exclusive skin and joint involvement. At the time of assessment, acquired organ damage was assessed using the SLICC/ACR Damage Index (SDI) and disease activity was assessed using the SLE Disease Activity Index 2000 (SLEDAI-2K) [28]. The majority (94%) of the patients had low disease activity or were in remission, as reflected by SLEDAI-2K scores of ≤ 5. Only 6% of the cohort displayed signs of active disease, although none of them had cardio-respiratory symptoms [29]. Vascular ultrasound was performed on 51/55 subjects (93%). Thirty-one sex- and age-matched healthy controls (HC) without known CVD or inflammatory conditions were also enrolled.

### Routine laboratory assessments

Plasma samples were collected and analysed for total cholesterol, triglycerides, high- and low-density lipoproteins, creatinine, C-reactive protein (CRP) with high sensitivity technique, and complement proteins C3 and C4 [30]. The level of creatinine was used to calculate the estimated glomerular filtration rate (eGFR) according to the MDRD 4-Variable Equation [31]. The presence of anti-double-stranded DNA (ant-dsDNA) antibodies was assessed using an addressable laser bread immunoassay (FIDIS™ Connective profile, Solonium software ver. 1.7.1.0; Theradiag, Croissy-Beaubourg, France) [32]. The analyses were performed at Linköping University Hospital.

### Trans-thoracic echocardiography

Comprehensive trans-thoracic echocardiography (TTE) was performed using the Vivid E95 ultrasound system (GE Healthcare, Chicago, IL, USA) with the 4Vc probe for assessments of cardiac morphology and function. All examinations were performed by two experienced sonographers. Measurements were performed off-line in the EchoPAC ver. 204 software (GE Healthcare) according to guidelines issued by the European Association of Cardiovascular Imaging and the American Society of Echocardiography [33, 34].

The LV end-diastolic volume and ejection fraction were measured using the semi-automatic quantification tool Auto-EF, and the left atrial end-systolic volume was determined using Simpson’s biplane method. Mitral E- and A-wave diastolic inflow velocities were measured by pulsed-wave Doppler *echocardiography* at the tips of the mitral leaflets, and early diastolic velocities (e' values) were measured by pulsed tissue Doppler *echocardiography* in the basal septum and lateral wall of the left ventricle. The E-wave to A-wave ratio (E/A) and the E-wave velocity to mean e’ velocity ratio (E/e’) were calculated. The right ventricular inflow diameter in end-diastole was measured in the right ventricular-focused four-chamber view. The systolic velocity in the right ventricular free wall (s’) was measured by pulsed tissue Doppler *echocardiography* in the basal right ventricular free wall, and tricuspid annular plane systolic excursion (TAPSE) was measured from M-mode registrations. A comprehensive evaluation of diastolic function was performed, as proposed by Tamas & Nylander [35].

**Fig. 1:**
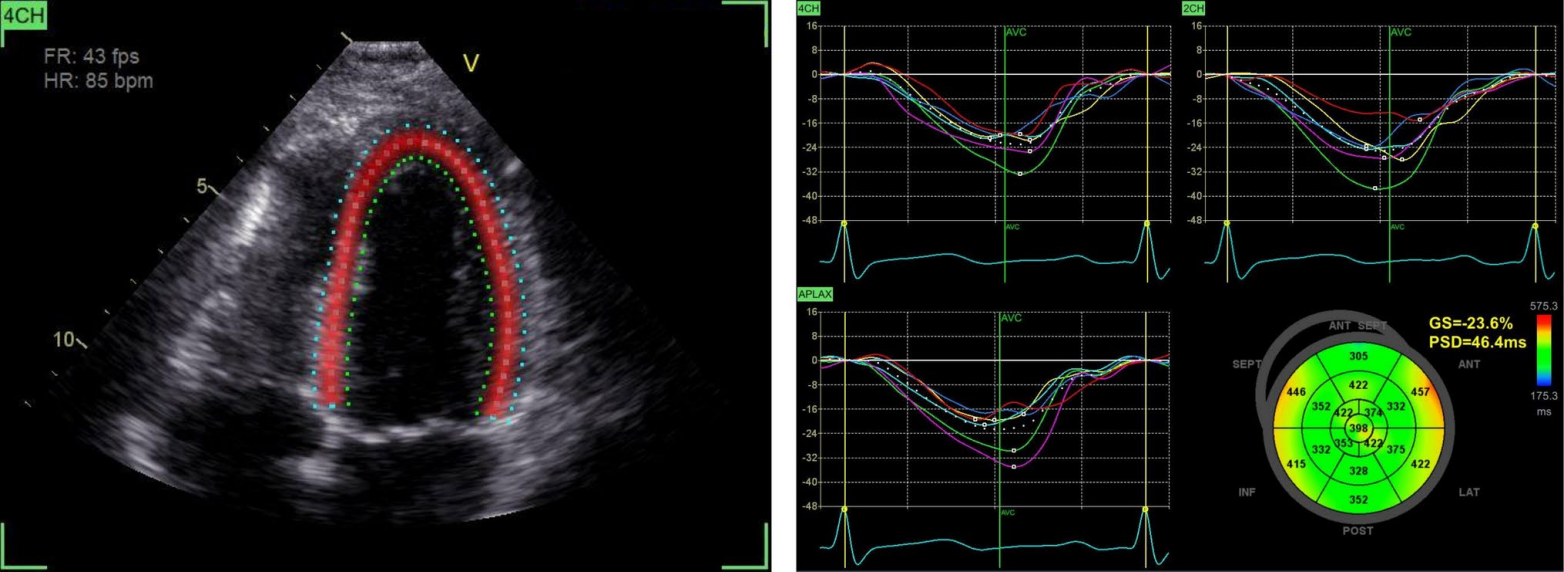
The left-side panel illustrate a speckle tracking echocardiography registration of the apical four chamber view. The right-side panel illustrates strain curves of longitudinal shortening and lengthening during the cardiac cycle, registered in the 18 left ventricular segments of the apical four, two and three chamber views, as well as a Bull’s eye plot depicting the time-to-peak longitudinal shortening of each segment. In this case, global longitudinal strain was -23.6% and mechanical dispersion, i.e. the standard deviation of time-to-peak longitudinal shortening for all segments, was 46.4 ms

The STE was performed using the Automated Functional Imaging tool in EchoPAC. Image quality was adequate for STE in all but five subjects. The GLS was calculated as the mean longitudinal strain of all LV segments from the apical four-, two- and three-chamber views, and LVMD was calculated as the standard deviation of the time-to-peak longitudinal strain for all the LV segments (Fig. 1). Right ventricular strain was measured in the right ventricular-focused four-chamber view. Global right ventricular strain was calculated as the mean longitudinal strain of all six right ventricular segments, and right ventricular free wall strain (RV FWS) was calculated as the mean longitudinal strain of the three right ventricular free wall segments.

### Vascular ultrasound

The carotid and central arteries were examined by using the GE Logic E9 Ultrasound system (LOGIQ E9 XDclear 2.0; General Electric Medical Systems Inc., Wauwatosa, WI, USA) with a linear transducer (L2–9 MHz). Intima-media thickness (IMT) and plaque occurrence in the carotid and central arteries, including the aortic arch, were evaluated by Duplex-ultrasound, as previously described [30]. Plaques were defined as focal areas of increased IMT > 0.5 mm or 50%, as compared to the IMT in the adjacent wall segment. All examinations were performed by the same experienced sonographer.

### Statistics

All statistical analyses were performed in the SPSS ver. 29.0.1.1 software (IBM Corp., Armonk, NY, USA). Continuous variables are presented as medians (interquartile range, [IQR]). Categorical variables are presented as absolute numbers (percent). For group comparisons, the independent samples *t*-test, Mann–Whitney *U*-test, one-way Analysis of Variance (ANOVA), Kruskal–Wallis one-way ANOVA or the Chi-squared (χ^2^) test were used, depending on the type and distribution of the data. Correlation analyses between IMT ICA, IMT aortic arch, and LVMD were performed using Spearman’s or Pearson’s methods, depending on the distribution of the data. Univariable and multivariable linear regression analyses were used to investigate associations between the degree of LVMD, clinical parameters, laboratory results and vascular disease. Variables with a p-value < 0.05 in the univariable analysis were included in the multivariable analysis. A p-value < 0.05 was considered statistically significant. Missing data were handled by list-wise deletion, to ensure robust statistical comparisons.

### Patient and public involvement

Neither patients nor the public were involved in the design, conduct, reporting, or dissemination of our research.

## Results

Detailed information on the demographics, laboratory data, and ongoing medical treatments for the 55 patients with SLE and the 31 HC is provided in Table 1.

**Table 1:** Detailed characteristics of the included patients with systemic lupus erythematosus (SLE) and the healthy controls (HC)

	SLE: APS (*n* = 18)	SLE: LN (*n* = 19)	SLE: Skin and joint (*n* = 18)	HC (*n* = 31)
Background variables				
Age at examination (years)	51.5 (40–61)	45 (38–52)	47 (35–59)	48 (38–59)
Female sex, *n* (%)	14 (78)	17 (89)	18 (100)	27 (87)
Duration of SLE (years)	18 (8–31)	12 (7–22)	11 (10–21)	N/A
SLEDAI-2K (score)	2 (0–2)	2 (0–4)	0 (0–2)	N/A
SLICC/ACR Damage Index (score)	1 (0–3)	0 (0–1)	0 (0–1)	N/A
Traditional risk factors and laboratory data				
Body mass index (kg/m^2^)	27 (24–30)	26 (24–30)	24 (21–29)	25 (22–27)
Ever smoker (former or current), *n* (%)	5 (28)	6 (31)	5 (29)	6 (19)
Diabetes, *n* (%)	2 (11)	1 (5)	0	0
eGFR (mL/min/1.73m^2^)	81 (74–87)	90 (82–90)	90 (81–90)	N/A
Total cholesterol (mmol/L)	4.8 (4.4–5.1)	4.1 (3.7–5.6)	4.8 (4.1–5.6)	N/A
Triglycerides (mmol/L)	1.1 (0.7–1.6)	1.2 (0.82–1.8)	0.9 (0.7–1.3)	N/A
HDL (mmol/L)	1.7 (1.3–2.0)	1.5 (1.1–1.8)	1.5 (1.3–2.0)	N/A
LDL (mmol/L)	2.5 (2.2–2.7)	2.2 (1.4–3.4)	3.0 (2.0–3.3)	N/A
Creatinine kinase (Ukat/L)	1.4 (0.99–2.7)	1.4 (1.3–2.0)	1.4 (1.0–1.65)	N/A
CRP (mg/L)	2.1 (0.8–4.0)	1.3 (0.7–4.5)	0.9 (0.5–1.3)	N/A
Complement protein C3 (g/L)	0.9 (0.79–1.1)	1.0 (0.8–1.2)	0.95 (0.84–1.1)	N/A
Complement protein C4 (g/L)	0.16 (0.11–0.22)	0.16 (0.11–0.2)	0.17 (0.12–0.20)	N/A
Anti-dsDNA (positive), *n* (%)	9 (50)	9 (47)	2 (11)	N/A
Anti-dsDNA (IU/mL)	43 (0–216)	0 (0–200)	0 (0)	N/A
IL-6 (above cut–off*), *n* (%)	3 (16)	1 (5)	3 (14)	N/A
IL-6 (ng/L)	2.5 (1.5–6)	1.5 (1.5–2)	1.5 (1.5–1.9)	N/A
Daily prednisolone dose (mg)	0 (0–5)	0 (0–5)	0 (0–2.5)	0
Ongoing medical treatments				
Anti-malarials	17 (94)	18 (94)	15 (83)	0
Anti-hypertensives	1 (5)	8 (42)	4 (22)	0
Glucocorticoids	10 (50)	10 (53)	4 (22)	0
Warfarin	11 (61)	1 (5)	0	0
Anti-platelet Therapy	1 (5)	4 (21)	0	0
Statins	4 (22)	2 (10)	2 (11)	0
Mycophenolate mofetil	3 (16)	7 (37)	1 (10)	0
Methotrexate	3 (16)	0	2 (10)	0
Leflunomide	0	0	1 (5)	0
Azathioprine	1 (5)	1 (5)	1 (5)	0
Sirolimus	1 (5)	0	1 (5)	0
Bortezomib	0	1 (5)	0	0
Belimumab	1 (5)	2 (11)	1 (5)	0
Intravenous Immunoglobulins	1 (5)	0	0	0

Data are presented as median (interquartile range) for continuous variables and *n* (%) for categorical variables

*APS* Antiphospholipid syndrome, *CRP* C-reactive protein, *dsDNA* double-stranded deoxyribonucleic acid, *eGFR* estimated glomerular filtration rate, *HC* Healthy Controls, *HDL* high-density lipoproteins, *IL* interleukin, *LDL* low-density lipoprotein, *LN* lupus nephritis, *N/A* not applicable, *SDI* Systemic Lupus International Collaborating Clinics/American College of Rheumatology Damage Index, *SLE* systemic lupus erythematosus, *SLEDAI-2K* Systemic Lupus Erythematosus Disease Activity Index 2000

* Cut–off: 7 ng/L

### Echocardiographic findings

**Fig. 2:**
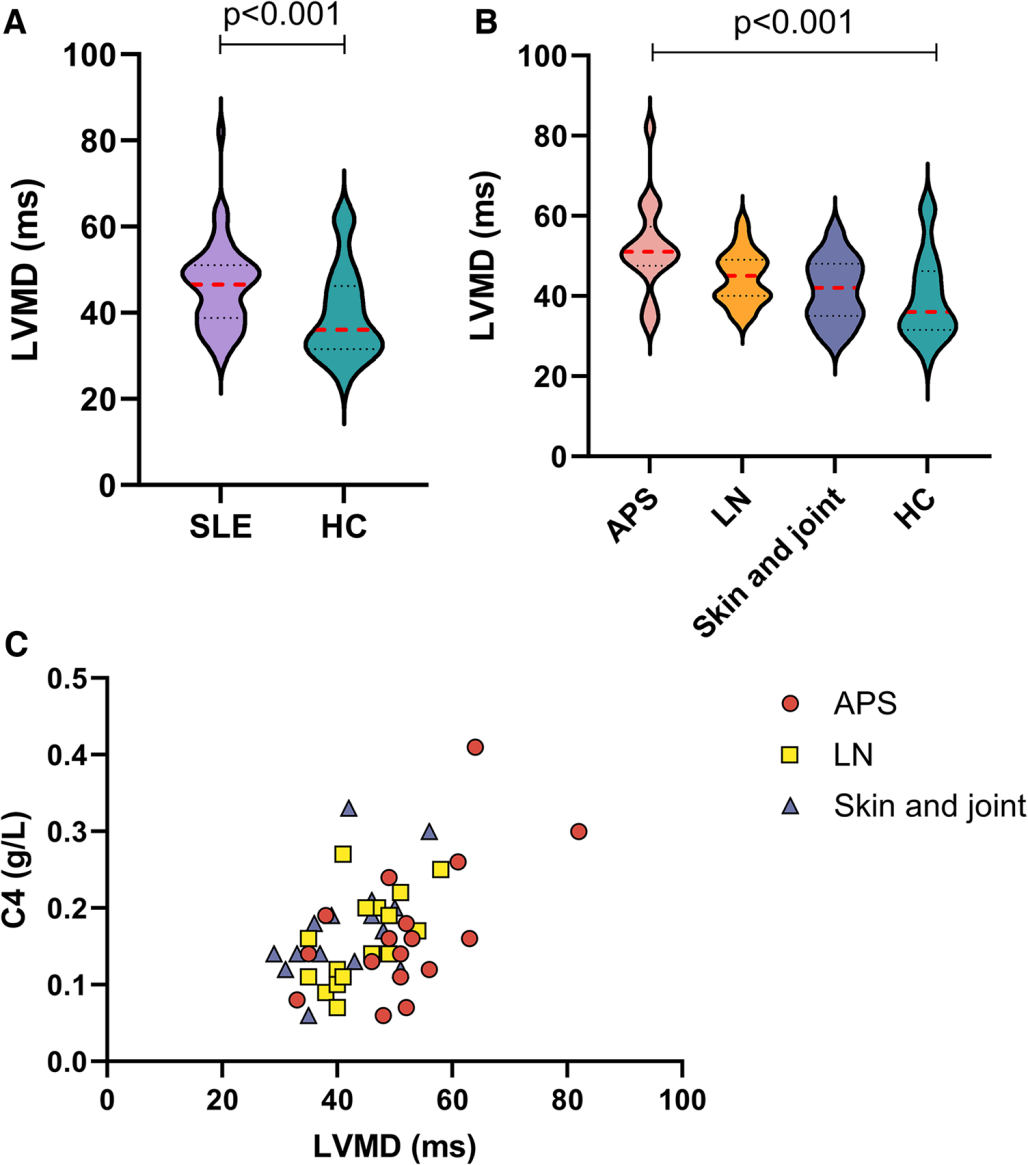
**A** Violin plot of left ventricular mechanical dispersion (LVMD) values in patients with systemic lupus erythematosus (SLE) and healthy controls (HC). The red dashed lines indicate the median values for each group. The LVMD is significantly higher in the SLE group than in the HC group (p < 0.001). **B** Violin plot of left ventricular mechanical dispersion (LVMD) values in the three SLE sub-groups and HC. **C** Scatter plot of complement C4 levels in the SLE sub-groups stratified by LVMD

The standard echocardiographic measures of cardiac morphology, LVEF and GLS did not differ between the SLE and HC groups, as shown in Table 2. Nevertheless, LVMD was significantly increased in the SLE cohort compared with the HC group (p < 0.001), with APS patients displaying the highest LVMD values (median, 51 ms) compared to the HC group (median LVMD, 36 ms) (p < 0.001). Patients with LN (median LVMD, 45 ms) and skin and joint involvement (median LVMD, 36 ms) did not differ significantly from the HC group (Fig. 2A, B). Furthermore, the patients with APS showed a slightly higher E/e’ ratio (p = 0.002), while measures of right ventricular function, i.e. TAPSE (p = 0.002) and RV FWS (p = 0.017), were slightly impaired in both the APS and LN groups in comparison to the HC group.

**Table 2:** Echocardiographic findings for the included patients with systemic lupus erythematosus (SLE) and the healthy controls (HC)

Echocardiographic Parameter	SLE: APS (*n* = 18)	SLE: LN (*n* = 19)		SLE: Skin and joint (*n* = 18)	HC (*n* = 31)	p-value
LEFT HEART:						
Morphological parameters:						
LV EDVI (ml/m^2^)	53 (43–62)	54 (46–62)		57 (50–69)	54 (50–62)	0.3
LA ESVI (ml/m^2^)	31 (21–40)	27 (21–35)		28 (24–32)	28 (23–33)	0.5
Functional parameters						
LVEF (%)	57 (55–60)	57 (56–61)		58 (57–61)	59 (58–60)	0.07
LV GLS (%)	– 19 (– 21 to– 19)	– 20 (– 21 to– 17)		– 21 (– 23 to– 20)	– 20 (– 21 to– 19)	0.4
LVMD (ms)	51 (48–57)	45 (40–49)		42 (35–48)	36 (32– 46)	** < 0.001**
E/A ratio	1.2 (0.8–1.6)	1.3 (1.0–1.7)		1.5 (1.2–2)	1.7 (1.2–2.0)	0.086
E/e’	7.6 (6–9)	6 (5–8)		6 (5–8)	6 (5–6)	**0.002**
Diastolic Function grade DF, *n* (%)	Normal 15 (83) Abnormal 3 (17)	Normal 14 (74) Abnormal 4 (26)		Normal 18 (100)	Normal 28 (90) Abnormal 3 (10)	0.18
RIGHT HEART:						
Morphological parameters:						
RVOT d (mm)	32 (28–35)	31 (27–34)		29 (26–33)	31 (28–34)	0.3
RV basal diameter (mm)	35 (30–38)	36 (31–38)		33 (31–36)	35 (32–36)	0.4
RAESVI (ml/m^2)^	19 (17–24)	18 (15–21)		21 (17–24)	21 (17–23)	0.4
Functional parameters:						
TAPSE (mm)	20 (17–22)	19 (16–21)		21 (19–25)	21 (21–24)	**0.002**
RV GLS (%)	– 22 (– 24 to– 21)	– 22 (– 24 to– 18)		– 25 (– 27 to– 21)	– 24 (– 26 to– 20)	0.07
RV FWS (%)	– 27 (– 28 to– 23)	– 26 (– 28 to– 22)		– 29 (– 32 to– 26)	– 29 (– 32 to– 25)	**0.017**

Data are presented as median (interquartile range) for continuous variables and *n* (%) for categorical variables

*APS* antiphospholipid syndrome, *E/A ratio* ratio of the early (E) to late (A) ventricular filling velocities, *E/e’* ratio of early diastolic mitral inflow velocity to early diastolic mitral annulus velocity, *LA ESVI* left atrial end-systolic volume index, *LN* lupus nephritis, *LVEF* left ventricular ejection fraction, *LV EDVI* left ventricular end-diastolic volume index, *LV GLS* left ventricular global longitudinal strain, *LVMD* left ventricular mechanical dispersion, *LVMI* = left ventricular mass index, *RA ESVI* right atrial end-systolic volume index, *RV basal diameter* right ventricular basal diameter, *RV FWS* right ventricular free wall strain, *RV GLS* right ventricular global longitudinal strain, *RVOT d* right ventricular outflow tract diameter, *TAPSE* tricuspid annular plane systolic excursion

LV diastolic function, assessed using the Decision Support tool developed by Tamas & Nylander [35], did not differ between the groups (Supplementary Table S1).

### Atherosclerotic plaque prevalence and intima-media thickness

Atherosclerotic plaques were detected in 26 (50%) of the subjects, predominantly located in the carotid bifurcation (58%). The subclavian/axillary artery and the common femoral artery also showed high frequencies of plaques (31% and 12%, respectively). The IMT values were normal in all the examined areas.

Patients were stratified based on LVMD values above or below the median (46.5 ms). The atherosclerotic plaque burden was markedly higher in the high-LVMD group (60% vs. 16%), as shown in Table 3.

**Table 3:** Characteristics of the patients with systemic lupus erythematosus with high or low LVMD values

Patients’ characteristics	LVMD > median of 46.5 ms (*n* = 25)	LVMD ≤ median of 46.5 ms (*n* = 25)	p-value
Age (years)	50 (40–60)	46 (33–51)	**0.033**
Female sex, *n* (%)	21 (84)	23 (92)	0.6
Body mass index (kg/m^2^)	26 (23–32)	26 (23–27)	0.18
Ever smoker (former or current), *n* (%)	10(40)	6 (24)	0.3
Systolic blood pressure (mm Hg)	130 (120–140)	125 (103–133)	**0.04**
Diastolic blood pressure (mm Hg)	80 (75–85)	80 (70–80)	0.3
Duration of SLE (years)	18 (8.5–30)	11 (8–18)	**0.02**
SLEDAI-2K (score)	0 (0–20)	0 (0)	**0.08**
SLICC/ACR Damage Index (score)	1 (0–3)	0 (0–2)	**0.008**
History of pericarditis, *n* (%)	4 (16)	2 (8)	1.0
Vascular status			
Plaque, *n* (%)	15 (60)	4 (16)	**0.04**
IMT ICA (mm)	0.45 (0.40–0.51)	0.42 (0.32–0.50)	**0.04**
IMT CCA (mm)	0.55 (0.51–0.66)	0.53 (0.50–0.62)	0.7
IMT SCA (mm)	0.57 (0.50–0.62)	0.55 (0.47–0.60)	0.1
IMT AxA (mm)	0.52 (0.45–0.60)	0.47 (0.40–0.55)	0.07
IMT CFA (mm)	0.55 (0.45–0.75)	0.51 (0.41–0.60)	0.07
IMT SFA (mm)	0.47 (0.40–0.52)	0.42 (0.35–0.47)	0.2
IMT aortic arch	1.25 (1.10–1.43)	1.0 (0.85–1.15)	** < 0.001**
Laboratory data			
eGFR (mL/min/1.73 m^2^)	84 (77–90)	90 (82–90)	0.2
CRP (mg/L)	2.1 (1.0–4.5)	0.9 (0.45–1.75)	0.07
IL-6 (ng/L)	2.2 (1.5–4.6)	1.5 (1.5–1.9)	0.3
Complement protein C3 (g/L)	1.1(0.88–1.2)	0.87 (0.80–0.99)	**0.014**
Complement protein C4 (g/L)	0.18 (0.14–0.24)	0.14 (0.11–0.19)	**0.044**
Total cholesterol (mmol/L)	4.9 (4.4–5.5)	4.2 (4–5)	0.177
Triglycerides (mmol/L)	1.1 (0.8–2.05)	0.88 (0.71–1.3)	**0.04**
HDL (mmol/L)	1.6 (1.3–2.0)	1.6 (1.3–1.9)	0.8
LDL (mmol/L)	2.6 (2.2–3.1)	2.4 (2–3)	0.5
Plasma albumin (g/L)	43 (40–46)	42 (40–44)	0.6
Urine albumin/creatinine ratio	0 (0–778)	0 (0–10)	0.2
Ongoing therapies			
Anti-malarials, *n* (%)	22 (88)	23 (92)	1.0
Statins, *n* (%)	4 (16)	2 (8)	0.4
Daily Prednisolone dose (mg/day)	2.5 (0–5)	0 (0–0.25)	0.2

Values are presented as median (interquartile range) for continuous variables and *n* (%) for categorical variables

*HDL* High-density lipoproteins, *IMT ICA* intima-media thickness internal carotid artery, *IMT CCA* intima-media thickness common carotid artery, *IMT SCA* intima-media thickness subclavian artery*, IMT AxA* intima-media thickness axillary artery, *IMT CFA* intima-media common femoral artery, *IMT SFA* intima-media thickness superficial femoral artery*, IMT aortic arch* intima-media thickness aortic arch, *LDL* low-density lipoprotein, *SDI* SLICC/ACR Damage Index, *SLEDAI-2K* Systemic Lupus Erythematosus Disease Activity Index 2000

A similar trend was observed for the IMT of the internal carotid artery (IMT ICA: 0.45 mm vs. 0.42 mm; p = 0.04) and aortic arch (IMT aortic arch: 1.25 mm vs. 1.0 mm; p = 0.001) (Table 3). In the overall SLE cohort, correlation analyses showed moderate, positive associations between the IMT ICA and LVMD (r = 0.34, p = 0.021), as well as between IMT aortic arch and LVMD (r = 0.45, p = 0.001).

### Predictors of LVMD

The univariable analyses revealed associations between LVMD and age, smoking history, SLE duration, disease activity (SLEDAI-2K), IMT ICA, IMT aortic arch, and complement protein concentrations (C3 and C4). In the multivariable regression analysis, age, SLEDAI-2K, IMT aortic arch and complement protein C4 level remained significantly associated with LVMD (Table 4). Relations between LVMD and C4 concentration in the three SLE phenotype groups are illustrated in Fig. 2C.

**Table 4:** Associations between clinical characteristics and left ventricular mechanical dispersion (LVMD) in patients with systemic lupus erythematosus

Characteristics	Univariable Analysis Standardized Coefficient Β (SE)	p-value	Multivariable Analysis^*^ Standardized Coefficient Β (SE)	p-value
Female sex, *n* (%)	– 0.08	0.5	—	—
Age (years)	0.3	**0.026**	0.2	**0.05**
Body mass index (kg/m^2^)	– 0.40	0.78	—	—
Ever smoker (former or current), *n* (%)	0.08	0.54	—	—
Systolic blood pressure, mmHg	0.2	0.07	—	—
Duration of SLE (years)	0.3	**0.006**	0.04	0.9
SLEDAI-2K (score)	0.2	**0.05**	0.27	**0.01**
SLICC/ACR Damage Index (score)	0.25	0.078	—	—
Plaque, *n* (%)	0.2	**0.05**	0.11	0.3
IMT ICA (mm)	0.3	**0.013**	0.06	0.5
IMT aortic arch(mm)	0.45	**0.001**	0.41	**0.001**
eGFR (mL/min/1.73m^2^)	– 0.122	0.4	—	—
CRP (mg/L)	0.224	0.118	—	—
Plasma albumin	0.009	0.953	—	—
IL-6 (ng/L)	0.24	0.09	—	—
Complement protein C3 (g/L)	0.44	**0.001**	-0.012	0.5
Complement protein C4 (g/L)	0.5	** < 0.001**	0.45	**0.006**
Urine albumin/creatinine ratio	0.059	0.685	—	—

*IL-6* interleukin 6, *IMT ICA* intima-media thickness internal carotid artery, *IMT aortic arch* intima-media thickness aortic arch, *SLEDAI-2K* Systemic Lupus Erythematosus Disease Activity Index 2000

^*^The multivariable analysis model included the following parameters: age, SLE duration, SLEDAI-2K, plaque occurrence, IMT ICA, IMT aortic arch, and complement proteins C3 and C4. The forward stepwise selection method was used in this model

## Discussion

Our study reveals numerous observations with significant novelty. First, we demonstrate that LVMD is significantly increased in patients with SLE, with the highest values being observed in individuals with concomitant APS, followed by those with renal involvement. The heterogeneous contraction pattern of the left ventricle observed for the patients with SLE with normal LVEF and normal GLS indicates sub-clinical myocardial disease. Second, our findings show that aberrant LVMD is associated with more-severe atherosclerosis and higher levels of complement protein C4. Third, SLE subjects with APS exhibited impaired LV diastolic function, as reflected by a slightly higher E/e’ ratio, and mild right ventricular impairment, as represented by lower TAPSE and RV FWS, in both APS and LN, suggestive of bi-ventricular affection.

Heterogeneous myocardial contraction, as evidenced by increased LVMD, is associated with myocardial fibrosis [36–38], and has recently attracted increased interest, as it has been found to predict adverse events, in particular ventricular arrhythmias, in patients with ischemic heart disease and various cardiomyopathies [14]. Little is known about LVMD in the general population, but increased LVMD is associated with older age, conduction disturbances and a more-severe CVD risk profile [39]. Indeed, patients with SLE carry a high CVD risk profile based on both traditional and non-traditional risk factors for atherosclerosis [40]. Our observation of a more-heterogeneous contraction pattern in SLE is similar to a previous study that included 35 subjects with SLE, reporting higher LVMD in patients who had a high-risk antiphospholipid antibody (aPL) profile despite exhibiting normal conventional echocardiographic parameters [41]. However, in contrast to our findings, He and colleagues reported increased LVMD in LN compared with other SLE phenotypes [21]. Notably, however, the latter study did not account for the aPL profile, and classified all other phenotypes as extra-renal SLE, potentially influencing the comparison.

The clinical relevance of increased LVMD in patients with SLE remains an area of active investigation [17, 22, 42]. In coronary artery disease and cardiomyopathies, LVMD is associated with worse clinical outcomes [14, 43]. However, whether this translates to a higher CVD risk in SLE requires further investigation. Two recent studies have shown a higher degree of LVMD among patients with SLE who have higher disease activity, positioning LVMD as a potential marker of sub-clinical myocardial dysfunction in patients with SLE [42, 44]. These findings are consistent with the results of our present study, which in a multivariable analysis identifies a significant association between SLE disease activity, assessed by SLEDAI-2K, and LVMD. In the context of clinical relevance, it is important to note that LVMD is easily analysed by semi-automatic and commercially available software.

A unique aspect of our study was the use of high-frequency ultrasound imaging of the carotids and the central arteries, including the aortic arch, which revealed a higher prevalence of atherosclerotic plaques, and higher IMT in the internal carotid artery and aortic arch in patients with increased LVMD. To the best of our knowledge, this association has not been previously explored. Previous studies utilised carotid ultrasound to assess atherosclerosis in SLE versus HC, demonstrating accelerated progression of carotid plaques in SLE [6, 45–47]. Our observations suggest that atherosclerosis contributes to sub-clinical myocardial dysfunction, as reflected by greater LVMD, even in the early stages when conventional echocardiographic parameters appear normal. The results warrant increased awareness among clinicians, leading to effective strategies for prevention of CVD events in subjects with SLE, e.g. lifestyle modification, control of disease activity and use of anti-malarials [40].

Previous studies have established an association between APS and LN with an increased burden of atherosclerosis, which is driven by endothelial dysfunction, immune cell dysregulation, autoantibodies, immune complex deposition, complement activation, and cytokine-mediated inflammation [48]. In our multivariable regression analysis, complement C4 emerged as a significant predictor of LVMD, which is a novel finding. Nevertheless, previous studies have examined the associations between CVD and the complement system [49]. A population-based prospective study from southern Sweden reported correlations between complement C3/C4 and CVD risk factors, with only C4 remaining independently predictive of CVD incidence [50]. Yet another relevant study identified baseline serum C4 levels as an independent predictor of stroke in patients undergoing coronary angiography [51].

Hypothetically, high C4 levels reflect inflammation in the atherosclerotic plaques in both the macro- and micro-vasculature, subsequently leading to myocardial dysfunction. This finding may appear paradoxical, as C3 and C4 levels are typically reduced in active SLE due to complement consumption [52]. However, in the present study, it was primarily subjects in remission or with low disease activity on stable immunosuppressive therapy who were included, which may explain the observed C4 levels above the lower reference limit. Similar results were seen in a study conducted by Mulvihill et al., who investigated complement components in childhood-onset SLE, finding higher C4 concentrations in patients with hypertension [53]. The mechanistic interplay between complement activation, atherosclerosis, and myocardial dysfunction warrants further investigation [54]. Several complement proteins, including C3, C5, and C1q, have been implicated in atherogenesis, suggesting a potential avenue for targeted therapies to mitigate CVD risk in the general population and specifically in patients with SLE [55]. To note, a great majority of the included patients in our study were prescribed anti-malarial agents which, apart from reducing thrombotic events, also have been ascribed cardioprotective effects in recent review articles [40, 56].

To our knowledge, this is the first study to assess systematically LVMD in well-characterised SLE sub-groups, including patients with APS, LN and skin and joint manifestations, all of which have distinct CVD risk profiles. The use of advanced STE enables sensitive detection of sub-clinical myocardial dysfunction, even in patients with preserved LVEF and GLS. Another strength of this study is the extended protocol of vascular ultrasound for plaques and IMT measurements.

Several limitations should be acknowledged. First, the relatively small sample size limits the generalisability of our findings and may have reduced the power to detect sub-group differences. Second, the cross-sectional design precludes causal inferences regarding the relationships between vascular and cardiac abnormalities. Third, whereas IMT and the presence of plaques serve as surrogate markers for atherosclerosis, the study includes no characterisation of the coronary circulation. The study focused on sub-clinical cardiac dysfunction and long-term follow-up is needed to assess whether the patients with SLE with increased LVMD are at higher risk of clinical cardiac events, such as arrhythmias and heart failure. Finally, we acknowledge as a limitation that data on some relevant CVD risk factors, e.g. family history and physical inactivity, were not available to us.

## Conclusion

SLE, and particularly in combination with APS, is associated with heterogeneous myocardial contraction indicative of sub-clinical myocardial disease. The associations of LVMD with plaque burden and IMT ICA suggest a contributory role of atherosclerosis in myocardial dysfunction, while the observed correlation with complement factors, particularly C4, highlights the underlying immunological mechanisms and potential directions for future research and therapeutic interventions.

## Supplementary Information

Below is the link to the electronic supplementary material.

Supplementary file1 (DOCX 16 KB)

Supplementary file2 (DOCX 28 KB)

## Data Availability

All data supporting the findings of this study are available within the paper and the *Supplementary Information*.

## Declarations

### Congress abstract publication

Preliminary data from the current work have been previously presented at one scientific meeting [57].
