# Supplementary material for: Heterogeneous myocardial contraction detected by speckle tracking echocardiography in systemic lupus erythematosus is associated with complement protein C4: a cross-sectional study from a Swedish tertiary referral centre
Source: Rheumatol Int. 2025 Aug 6;45(8):183. doi: 10.1007/s00296-025-05939-8 (PMC12328509; doi:10.1007/s00296-025-05939-8)
Supplement: Supplementary file 2 — Supplementary file2 (DOCX 28 KB) [file 296_2025_5939_MOESM2_ESM.docx]

# Supplementary material

## Supplementary Table S1. Left ventricular diastolic function status in the SLE sub-groups and in healthy controls.

| **LV Diastolic Function** | **APS**  (*n* = 18) | **LN**  (*n* = 19) | **Skin and joint**  (*n* = 18) | **HC**  (*n* = 31) | **p-value** |
| --- | --- | --- | --- | --- | --- |
| Normal function | 13 (72.2%) | 14 (73.7%) | 18 (100.0%) | 28 (90.3%) | 0.21 |
| Grade 1 dysfunction | 3 (16.7%) | 4 (21.1%) | 0 (0.0%) | 3 (9.7%) |  |
| Grade 2 dysfunction | 0 (0.0%) | 0 (0.0%) | 0 (0.0%) | 0 (0.0%) |  |
| Grade 3 dysfunction | 2 (11.1%) | 0 (0.0%) | 0 (0.0%) | 0 (0.0%) |  |
| Missing | 0 (0.0%) | 1 (5.3%) | 0 (0.0%) | 0 (0.0%) |  |

**APS**, antiphospholipid syndrome; **HC**, healthy controls; **LN**, lupus nephritis. Assessments based on the decision support algorithm described by Tamas *et al*. (https://diastolicfunction.it.liu.se/Home/English).
